# Intelligent Electromagnetic Sensors for Non-Invasive Trojan Detection

**DOI:** 10.3390/s21248288

**Published:** 2021-12-11

**Authors:** Ethan Chen, John Kan, Bo-Yuan Yang, Jimmy Zhu, Vanessa Chen

**Affiliations:** Department of Electrical and Computer Engineering, Carnegie Mellon University, Pittsburgh, PA 15213, USA; johnkan@andrew.cmu.edu (J.K.); boyuany@andrew.cmu.edu (B.-Y.Y.); jzhu@cmu.edu (J.Z.); vanessachen@cmu.edu (V.C.)

**Keywords:** hardware security, electromagnetic sensing, machine learning, real time

## Abstract

Rapid growth of sensors and the Internet of Things is transforming society, the economy and the quality of life. Many devices at the extreme edge collect and transmit sensitive information wirelessly for remote computing. The device behavior can be monitored through side-channel emissions, including power consumption and electromagnetic (EM) emissions. This study presents a holistic self-testing approach incorporating nanoscale EM sensing devices and an energy-efficient learning module to detect security threats and malicious attacks directly at the front-end sensors. The built-in threat detection approach using the intelligent EM sensors distributed on the power lines is developed to detect abnormal data activities without degrading the performance while achieving good energy efficiency. The minimal usage of energy and space can allow the energy-constrained wireless devices to have an on-chip detection system to predict malicious attacks rapidly in the front line.

## 1. Introduction

**Figure 1:**
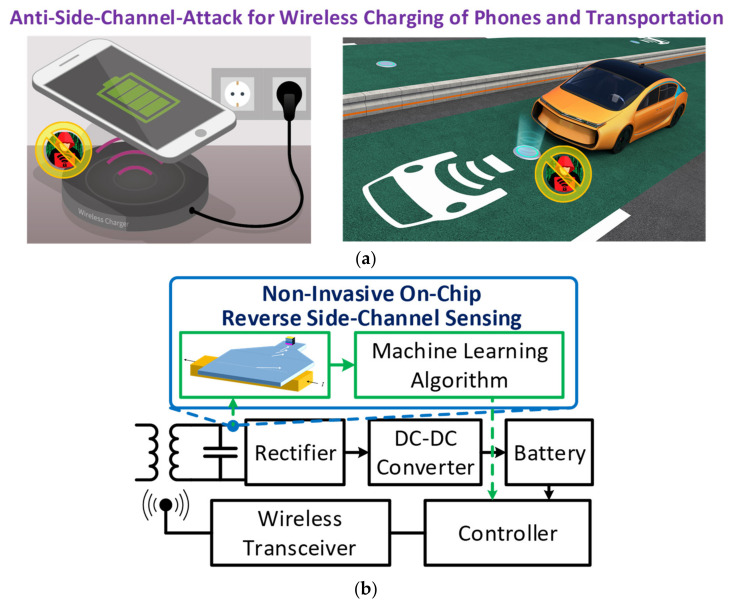
(**a**) Security vulnerability of electromagnetic emissions and wireless charging (figure credit for wireless charging: Infineon and PowerElectronics.com Available online: https://www.powerelectronics.com/markets/automotive/article/21864097/wireless-charging-of-electric-vehicles accessed on 9 December 2021), and (**b**) the proposed non-invasive on-chip sensing system.

The rapid growth of sensors and the Internet of Things (IoT) has the potential to transform society, the economy, and the quality of life. Many devices at the extreme edge collect and transmit sensitive information wirelessly for remote computing. The sensitive information can be leaked from side channels, including power consumption and electromagnetic (EM) emissions. Some devices are simply controlled by a simple wake-up signal to activate data transmission without two-way authentication. Moreover, the wireless charging techniques that allow energy constrained devices and electric cars to stay connected and operate continuously provide another entry point to exploit the sensitive information and the vulnerability in the power domain, as shown in Figure 1a. The vulnerability of those wireless devices to hacking or exploitation has emerged as a major concern on both security and public safety. For instance, because the electronic devices may continue receiving and transmitting signals while they are being wirelessly powered, the data activities are exposed to the energy source. Nevertheless, state-of-the-art cybersecurity approaches are mainly focused on software and digital modules. Security measures are not integrated in the analog/radio frequency (RF) domain to verify signal and power sources or to suppress the side-channel emissions in real time. To bridge the gap, this study presents a self-testing approach incorporating nanoscale EM sensing devices and learning algorithms to detect threats directly at the RF and analog front-end. As shown in Figure 1b, the EM sensors are integrated into the RF/analog front-end through post processing to monitor the EM emissions from power wires and critical signal nodes. Machine-learning modules were developed to analyze the sensed data for threat and vulnerability detection. Combing emerging material, device, circuit, and system concepts, this study developed a built-in threat detection approach in the RF/analog domain without degrading the performance while achieving good energy efficiency.

## 2. Relevant Work

Wireless power transfer technology relies on the principle of electromagnetic induction, which falls into two categories, near field and far field. Near-field techniques utilize inductive coupling between coils of wire or capacitive coupling between metal electrodes [1,2,3]. Inductive coupling for power transfer over a short distance through magnetic fields is one of the most widely used wireless powering technologies. Considering the conversion efficiency and the safety criteria, radiative wireless power transfer is the most popular far-field technique to remotely power mobile devices over a long distance for low-power devices [4,5,6] and wireless sensor networks (WSNs) [7]. Nondirective antennas can be used to energize sensors, but the efficiency is low. On the other hand, directive transmitting antennas are more efficient to increase the maximum distances that can be remotely powered [8]. Many radiative wireless power transfer techniques have been discussed with different operation frequencies, average chargeable distances, beamforming techniques, and overall system complexity. Depending on different transfer schemes, specific wireless power transfer patterns can be adopted to charge the devices without interrupting the operations. For instance, the electric vehicles can be dynamically charged as they ride on the wireless power lane [9]. Hence, the wireless power source is becoming a new shared input to the devices and vehicles as they are all connected on the wireless charging platform, so the data activities are exposed to the energy source. Especially when the devices and vehicles with hardware trojans need to be recharged, its battery can be very low and the existing security functions may not work intermittently, which raises critical concerns of safety and security. However, the security vulnerabilities for pervasive devices accessing the shared wireless charging platform have not been addressed.

### 2.1. Survey of Hardware Trojans

Much attention has been focused on hardware trojan taxonomy, development, and detection in the past two decades, especially since the Defense Science Board of US Department of Defense released a report in 2005 on the security of the supply of high-performance integrated circuits (ICs) which highlighted the need for “secure and authentic hardware” [10]. The resulting research produced numerous publications [11,12,13,14,15,16,17,18,19,20,21,22,23,24,25,26,27] which not only provide insight into existing hardware trojans, but also develop a general framework of hardware trojan understanding. This section will first briefly review hardware trojan taxonomy and detection methods, point out the lack of literature related to analog trojan development, taxonomy, and detection, and then present a number of trojans scenarios that can be possible in the analog/RF domain, specifically attacking a Class-E amplifier in the later sections.

#### 2.1.1. Hardware Trojan Taxonomy and Insertion

Hardware trojans defined by [12] are an intentional and malicious modification of a circuit that is designed to alter the circuit’s behavior in order to accomplish a specific objective. It also makes a distinction between such trojans and design bugs and defects by defining such a bug as “an unintentional problem (i.e., error) that is unknowingly introduced into the circuit during its design and development phases” and a defect as “unintentional physical phenomenon (e.g., imperfection) that occurs during the circuit’s fabrication, assembly, and testing phases”. Trojans, as they are malicious, seek to tamper with the function of the integrated circuit and avoid detection, whereas flaws and bugs are generally discoverable via conventional models of testing and verification.

Reference [11] provides an excellent description of a general view of hardware trojan taxonomy. Furthermore, it details the supply chain and hardware development layout for ASICs and FPGAs. Other resources, such as reference [12], also provide excellent insight into the taxonomy of hardware trojans and the section of the design process into which hardware trojans can be inserted. Hence, it is clear from the various literature that the number of trojans, their triggers, payloads, and development can be inserted in numerous areas of the design phase. These publications indicate not only the type of trojan trigger and payload, but also the potential points at which the trojan can be inserted [1,4,17] which all developed trojans that can be inserted by an untrusted foundry, or which insert the trojan post-fabrication [11,12].

However, while a number of papers indicate hardware trojans, most focus on the digital domain, even while utilizing “analog” trojans. A search of literature generally yields results in which papers such as [15,18] developed “analog” trojans. However, the in these papers, the circuits they are trying to attack generally are in the realm of digital integrated circuits. In [12] the authors indicate there are at least four types of trojans, side channel, semiconductor, analog, and digital, and includes a catch-all category of other for those that do not directly fall into those other categories. Side-channel attacks generally leak information out of an analog channel [12,14]. Semiconductor trojans generally tamper with the dopant polarity [17], analog trojans seek to insert some sort of analog device or additional circuit that will affect some sort of change in the operation of the circuit, either immediate or over time [14,20,21,22,23], such as the capacitor trojan threat identified in [18]. Digital trojans generally try to cause issues with the finite state machines (FSM) [12] to cause the overall FSM to end up in a “don’t-care” state, if available, that would contain a trojan payload.

Whilst there is a great deal of literature seeking to define hardware trojans, all of them, however, seek to attack circuits that remain in the digital domain. All the literature mentioned above, even those considered “analog” or even “semiconductor” trojans, seek to attack either microprocessors, digital circuits, etc. Additionally, some developed trojans for “RF applications”, but their trojans generally remain in the realm of different transmitter input termination [20], and do not cover the full spectrum of possible hardware trojan attack vectors in the RF domain. Hence, we seek to contribute not only to this prior research, but also to provide some initial steps at the lack of research into trojans that can occur in the analog domain.

#### 2.1.2. Analog Circuit Trojans

Hence, after noting the expansive literature focusing much needed attention on hardware trojans in the digital circuit design domain, the following papers attempt to address trojans in the analog circuit design domain. Quite a few indicate the noticeable lack of research in this field [23,25]. These trojans are more difficult to obfuscate or deceptively insert than larger, more topographically complex digital circuits that can feasibly hide a trojan, digital, analog or semiconductor, these trojans can still exist [25]. The class of power/area/architecture and signature transparent (PAAST) trojans impact the fundamental operation of analog circuits, such as amplifiers, but do not require extra components, area, or semiconductor changes [24]. The study states that by changing the high side supply bus to an amplifier or oscillator circuit, it is possible to cause the circuit to go into a trojan state without adding extra components. It also proves that even without having to physically change the circuit, hardware trojans within analog IC development exist. The research into PAAST trojans has generally focused on the determination of the possible trojan states [26].

Other trojans in the analog/RF front-end domain have also been detected such as changing the termination to the entrance of the power amplifier (PA) [20], or inserting a trojan on a mm-wave RF PA matching network to leak information [27]. Hence, while hardware trojans are possible, there has been little focus on detecting and defending the RF front end from inserted hardware trojans.

### 2.2. Hardware Trojan Detection

Hardware trojan detection methods are broadly categorized as destructive and nondestructive [11,12]. Destructive methods can be applied on a smaller scale, but also provide a way to find and form golden models for verification [12]. Nondestructive methods include techniques such as side-channel analysis, formal verification, simulation, and logic testing. Many nondestructive methods require a golden model, and thus, together, these nondestructive and destructive methods form a complementary approach to hardware trojan detection. Other recently studied methods include an optical analysis of various ICs [28].

#### 2.2.1. Side-Channel Detection

Side-channel analysis is a well-studied detection method, with physical side channels such as temporal (propagation delay), thermal, and electrical (current, EMI, voltage, charge). Side-channel attack (SCA) analysis utilizes the hardware runtime characteristic, such as power, of a cryptographic device to evaluate if it leaks secret information or reveals encryption behaviors. Unlike exploiting software bugs, such attacks on hardware components are not due to buggy hardware. Side-channel attacks can be categorized in a simple power analysis [29], differential power analysis [29], and correlation power analysis [30]. Since a correlation power analysis requires far fewer traces for recovering the key than a simple power analysis or differential power analysis, a correlation power analysis that retrieves the key through analyzing the correlation between the computing data and the measured power consumption, has become the most popular way for side-channel attacks to crack many cryptographic implementations [31,32]. Among many kinds of targets, an awareness of the potential of the EM side-channel attacks is developing [33,34,35,36,37,38,39]. The attacker is typically interested in emanations resulting from data processing operations, such as state changes and current flows in the CMOS circuits. These currents result in EM emanations, sometimes, in unintended ways. Such emanations carry information about the data or clock rates. The emanations provide multiple views of events unfolding within the device at each clock cycle because each active component produces and induces various types of emanations, increasing their vulnerability to hacking or exploitation. However, much of the literature on the utilization of current and EM side channels is generally not isolated from the side-channel under test [20], and it requires components to be placed in the circuit itself to detect changes in the waveform. In the case of an EM side-channel analysis, the unit under test must be within a particular test setup in order for those verifying the chip to discover the trojan, and once it leaves that setting, if the trojan goes undetected, it cannot be discovered until malicious events occur. Hence, this study proposes IC power interconnect the EM side-channel analysis via magnetic tunnel junction sensors.

#### 2.2.2. IC Current Sensing for Hardware Trojan Detection

Various IC current sensing methods have been previously proposed, including built-in current sensors (BICSs) [40] and magnetoresistance sensors [41,42]. Previously, BICS were employed and shown to be able to detect trojans [40]. Other research indicated that MTJ sensors can be utilized for anomaly detection [43]. In many current sensing schemes, the conventional methods utilize invasive series components, such as a series resistor, a power MOSFET (to observe on-resistance), and even an integrator [44,45,46,47]. These schemes cause high-power dissipation and have many limitations, including process dependency, control difficulty and high complexity. The major issue is that the inserted components change the characteristics of the overall circuits unless a small resistance component is inserted in the loop. Although using a small resistance component can reduce the risk of degrading the performance, it increases the difficulty to sense the signal accurately. In this study, novel on-chip non-invasive EM sensors will be exploited to collect EM emanations for (1) observing if the device reveals detectable patterns; (2) monitoring if the device is under attack, which may result in unusual activities. To enable the on-chip security detection function for mobile devices, we propose an EM-sensing system to monitor critical signals with non-invasive sensors that can avoid inserting new components in the signal path, so that the system characteristics will not be modified by the sensing circuits.

This study proposes to utilize MTJ sensors for current sensing, and machine learning models to develop an on-chip, isolated current sensor that will enable hardware trojan detection for protection of RF transceivers. Thus, this study not only focuses on the physics of hardware transceivers, computationally light-weight machine learning models, and MTJ sensors, but also develops a number of potential hardware trojans that cover the vulnerabilities pointed out in previous research, such as added components and injected noise.

## 3. On-Chip Magnetic Tunnel Junction (MTJ) Based Sensors for Instant Device Power/Current and EM Emission Monitoring

The basic MTJ structure consists of two ferromagnetic layers separated by the insulator layer. The pinned layer has the fixed magnetization direction, while the magnetization direction can be changed in the free layer. Conventionally, the MTJ devices have been used as oscillators or memory [48,49,50,51,52]. The MTJ devices can be fabricated monolithically over CMOS circuits. An e-beam-based nanofabrication process was developed to fabricate the MTJ-based spin torque oscillator over the Metal-4 layer of CMOS circuits. In this study we directly changed the resistance of MTJ devices with an external magnetic field. Hence, the MTJ devices are utilized as a non-invasive current sensor, which resistance is a function of the external magnetic field. The MTJ devices can be exploited as EM sensors placed near the critical signal paths.

**Figure 2:**
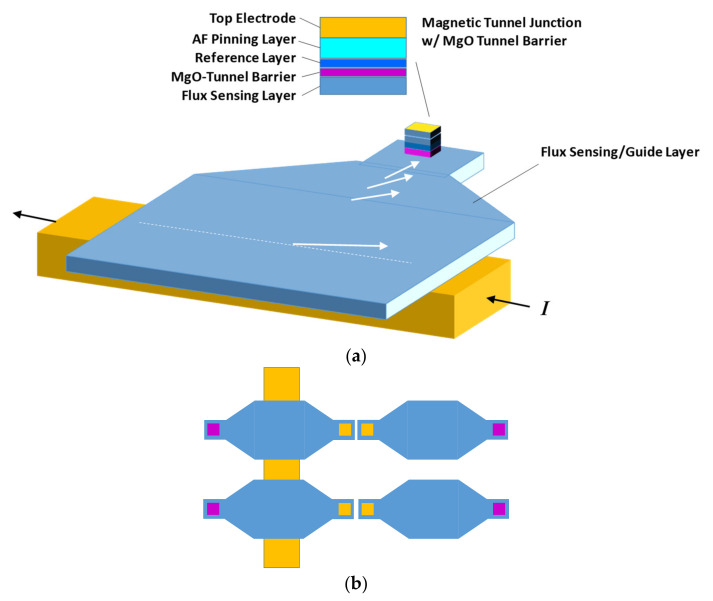
(**a**) The on-chip non-invasive current sensor that consists of the magnetic flux guide and concentrator along with magnetic tunnel junction, and (**b**) the bridge sensing structure to eliminate any response to field disturbance.

Figure 2a shows the on-chip non-invasive current sensor that consists of the magnetic flux guide and concentrator along with the magnetic tunnel junction to convert magnetization rotation into a voltage change. A current along the power line of the chip generates magnetization rotation in the above magnetic layer with its rotation magnitude linearly proportional to the current amplitude. The patterned planar funnel-shaped magnetic film will amplify the rotation angle as the magnetization flux travels along the strip. An MTJ is placed at the end of the strip with its free layer exchange coupled to the flux guide. An MgO-based tunnel barrier is used to obtain high magnetoresistance ratio (MR) of larger than 300%. The reference magnetic layer on the other side of the tunnel barrier has its magnetization pinned in the direction orthogonal to the flux propagation direction by using an antiferromagnetic layer deposited above. The resistance of the MTJ depends on the relative magnetization orientation of the two magnetic layers sandwiching the tunnel barrier, i.e., the angle *q* in the figure on the left. The resistance can be computed by $R\left(\theta \right)=\frac{R_{⊥}}{1+p^{2}\cos\theta }$ where *R*_⊥ is the resistance when *q* = 90° and *p* is polarization factor. The maximum and minimum resistance can be calculated as $R_{min}=R\left(0^{{}^{\circ}}\right)=\frac{R_{⊥}}{1+p^{2}}$ and $R_{max}=R\left(180^{{}^{\circ}}\right)=\frac{R_{⊥}}{1-p^{2}}$. Therefore, $MR=\left(R_{max}-R_{min}\right)/R_{min}=\left(\frac{R_{⊥}}{1-p^{2}}-\frac{R_{⊥}}{1+p^{2}}\right)/\frac{R_{⊥}}{1+p^{2}}=\frac{2p^{2}}{1-p^{2}}$. For today’s typical MTJ, *p* is equal to 0.70~0.75 and MR is equal to ~200%. The resulting resistance-area product $\left(R_{⊥}A\right)$ is around $1\;k\Omega \cdot {\mu m}^{2}\sim 1\;M\Omega \cdot {\mu m}^{2}$. The analysis shows that millivolts level signal voltage is expected for a milliampere-level current change. Here, a bridge sensing structure [53] in Figure 2b is used to eliminate any response to the external stray field disturbance, such as the earth field effect.

The entire MTJ-based sensor structure can be directly fabricated on top of the top metal layer of the semiconductor chip/circuit with two potential methods. The first one is the chemical mechanical polishing (CMP) process that will be performed over the top metal layer with deposition of the magnetic flux guide and MTJ film stack using the sputtering technique. An e-beam/optical lithography with an ion-mill process will be employed to fabricate the sensor structure along with contacting pads and connection to the circuit underneath. The other method is to adopt the dry etch to remove the top passivation layers for chip protection from the electrode areas. The silicon dioxide can be further thinned down by an optional dielectric reactive etch in order to enhance the coupling efficiency and the minimum detectable resolution.

## 4. Machine Learning Algorithms for Real-Time Threat-and-Vulnerability Detection

A typical side-channel signal analysis involves pre-processing to diminish dimensionality, where the measured traces are compared with predicted leakage using distinguishing algorithms. The most common technique is correlation computation [11]. For example, the Pearson correlation coefficient, ρ, for the information component, *t*, of all measured traces between predicted leakage, *L_p_*, and measured leakage, *L_m_*(*t*), is defined as follows: $\rho \left(t\right)=Cov\left(L_{p},L_{m}\left(t\right)\right)/\sqrt{Var\left(L_{p}\right)\cdot Var\left(L_{m}\left(t\right)\right)}$, where Cov is covariance and Var defines variance. Pre-processing is adopted to diminish the set of points in the trace to remove high-order signals. However, it is still computationally expensive to realize pre-processing and correlation computation on energy-constrained RF/analog devices. To eliminate the need of pre-processing the data, Bayesian neural networks (BNNs) are exploited to directly process data and extract the features in the proposed research.

### 4.1. Bayesian Neural Networks

Bayesian neural networks (BNNs) have been investigated as a computationally lightweight yet robust approach to the classification of electrical signals. In particular, a previous work [26] investigated the use of BNNs as a way to classify power amplifiers (PAs) based upon variational differences due to process corners. This study also investigated classifying side-channel signals sensed from the MTJ sensors, such as integrated circuit (IC) supply current, through Bayesian neural networks. BNNs are based upon Bayes’ probability theorem which states that the probability for a hypothesis from a given set of data D to be true is equal to the probability that D is true given a hypothesis h multiplied by the probability the hypothesis is true divided by the probability of *D*.

(1) $$P\left(h|D\right)=\frac{P\left(D|h\right)P\left(h\right)}{P\left(D\right)}$$

In this case, $P\left(h|D\right)$ is the posterior probability of *h* because it reflects the confidence that *h* holds after seeing *D*. Bayes concept learning is based upon some main assumptions, that is, that the BNN is trained utilizing a sequence of training examples (D), consisting of a set of instances x, which are mapped to a label, *y* such that:

(2) $$D=[\left(X_{n},y_{n}\right)|n=1,\;2,\;\ldots ,\;N]$$

For some *n*, $X_{n}\;$is a vector of a set of points corresponding to an IC current signal sensed through a MTJ resistive sensor and$\;y_{n}$ is a vector of assigned class labels, corresponding to a set of K classes. Given a model with parameters θ, and prior distribution$\;Pr\left(\theta \right)$ the posterior distribution for the parameters is as follows:

(3) $$\mathrm{Pr}\left(\theta |X_{tr},y_{tr}\right)=\frac{\mathrm{Pr}\left(\theta \right)\mathrm{Pr}\left(y_{tr}|X_{tr},\theta \right)}{\int Pr\left(\theta \right)Pr\left(y_{tr}|X_{tr},\;\theta \right)d\theta }$$

In classifying a test set $X_{new}$, the predictive distribution of the classification set $Y_{new}$ becomes:

(4) $$Pr\left(Y_{new}|X_{new},\;X_{tr},y_{tr}\right)=\int Pr\left(Y_{new}|X_{new},\;\theta \right)Pr\left(\theta |X_{tr},y_{tr}\right)d\theta$$

**Figure 3:**
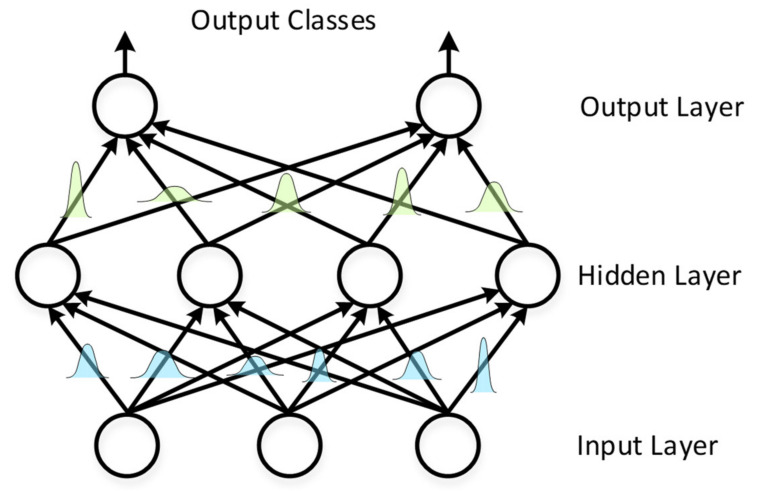
The weights of the Bayesian neural network weights are sampled from probability distributions.

Due to the intractable nature of the integral in Equation (4), various numerical methods, such as the computationally heavy Markov Chain Monte Carlo method, must be applied to estimate the predictive distribution. In this study, the comparatively lighter computational method variational inference [54] is used to estimate the integral. The variational posterior is assumed to be a Gaussian distribution, where the samples of the weights are obtained by shifting and scaling unit Gaussian variables with mean μ and standard deviation σ, where $\sigma =\ln\left(1+\exp\left(\rho \right)\right)$. Thus, each sample of the weights can be expressed as:

(5) w=μ+σ ∘ϵ

where “∘” denotes an element-wise multiplication and ϵ is a vector of Gaussian normal distribution $N\left(0,1\right)$ to introduce variance to the weights for the Bayesian neural network as in Figure 3.

### 4.2. BNN Architecture and Optimization

**Figure 4:**
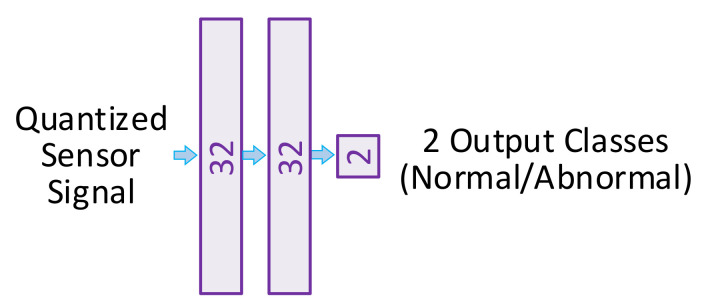
The optimized architecture of the lightweight Bayesian neural network for classification of the sensed EM signals.

The BNN for this study was a network of two hidden layers with thirty-two nodes per layer, as shown in Figure 4. The BNN was trained utilizing the Python library Pytorch. The Cadence simulation data were quantized and classified to train the BNN. The BNN was trained and tested with the data from a number of different hardware trojans. We first tested the ability of the system to classify individual trojans, the details of which will be discussed in a further section. For each of these cases, certain trojans were easier to detect than others, with some of the particular trojans being able to be detected with nearly 100% accuracy. Furthermore, we also trained and tested the BNN with all the different hardware trojans combined into one dataset. We equally trained the BNN with normal and abnormal data, and noticed that due to similarities between the normal and abnormal data, the accuracy for the overall combined dataset was around 90%. We determined the exact structure of our BNN in order to maximize the accuracy for the total trojan dataset and we found that utilizing 32 hidden neurons per layer produced nearly 6% higher accuracy after 1000 training epochs than 16 neurons, but more statistically insignificant accuracy depreciation than a network with 64 neurons in the same amount of time. Thus, we decided to utilize 32 neurons to minimize resource usage and accuracy.

## 5. Experimental Results

### 5.1. Fabracation of the MTJ Sensors

This work aims to develop the reliable methods for design and fabricating the novel MTJ sensor on the CMOS circuits. The planar funnel-shaped magnetic film was developed to efficiently amplify and convert the sensed magnetic field to a voltage change. The behavior of the MTJ sensor was characterized and modeled for the Cadence simulations.

#### 5.1.1. Fabrication

**Figure 5:**
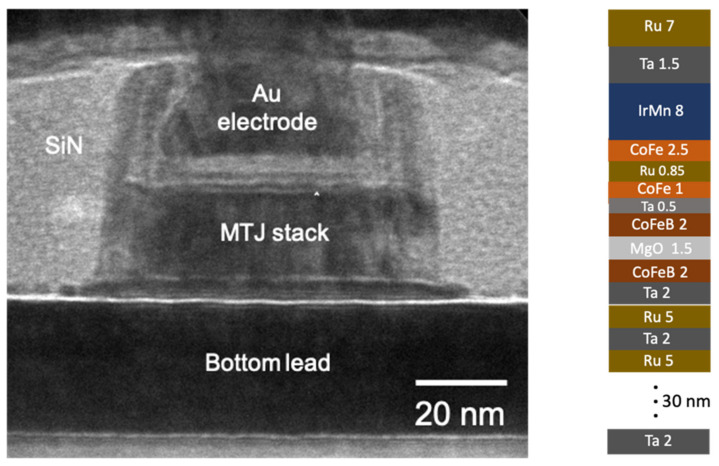
Cross-section TEM images of the MTJ pillar and the film structure.

To fabricate MTJ on top of top metal layer, first, we used chemical-mechanical planarization (CMP) to polish and planarize the passivation layer. Then we deposited the bottom electrode and MTJ stack at room temperature by magnetron sputtering with base pressure <2 × 10^−8^ Torr. The film structure, as shown in Figure 5, is Ta/Ru multilayer (30)/CoFeB(2)/MgO(1.5)/CoFeB(2)/Ta(0.5)/CoFe(1)/Ru(0.85)/CoFe(2.5)/IrMn(8)/Ta(1.5)/RU(7) (in nm). After deposition, the film is post-annealed at 330 °C for 10 min with a 5000 Oe magnetic field applied along in the plane direction. The deposited film is processed into elliptical pillars by e-beam lithography and carefully controlled ion milling. The long and short axis of the pillars are 300 nm and 70 nm, respectively. We deposited a SiN layer on top of the nanopillars for passivation followed by low angle ion milling for planarization. The trench and via were defined by photolithography and etched by reactive ion etching (RIE). Finally, Ti(5)/Au(100) (in nm) was deposited for the top electrode. Figure 5 also shows the cross-section image of the MTJ device.

#### 5.1.2. MTJ Measurement Results

**Figure 6:**
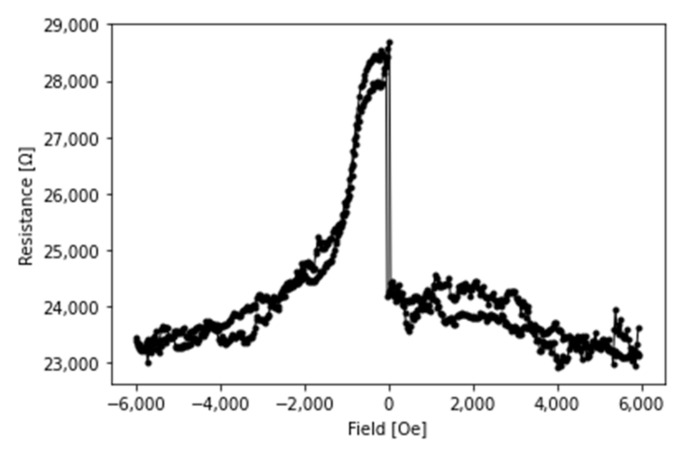
Typical tunneling magnetoresistance (TMR) curves. This graph illustrates the H-field-resistance curve for one MTJ sensor developed for this study. Two sweeps of the H-field are separate tests of the sensor. For the purposes of our modeling, we used the greater resistance response.

The MTJ sensor is characterized by applying magnetic field along the axis of the pillar and measured the resistance change corresponding to the magnitude of magnetic field. Figure 6 shows the typical tunneling magnetoresistance (TMR) curves. The sensor is in the low-resistance state when the two CoFeB layers’ magnetizations are aligned in the parallel state by a large magnetic field. While in the range of a small magnetic field, the magnetization of the sensing CoFeB layer with respect to the reference CoFeB changes gradually with the field intensity and eventually reaches a high-resistance state when they are in the antiparallel state.

#### 5.1.3. Modeling for Cadence Simulation

The measured results discussed in Section 5.1.2 were utilized to find the relationship between the magnetic field and resistance for this particular MTJ resistor. Once the magnetic field-resistance relationship was characterized, then electromagnetism, in particular Ampere’s law, could be used to find the current-resistance relationship of the sensor.

The numerical analysis indicated the relationship between the magnetic field and resistance was piecewise linear in two different regions of interest:

(6) $$R=\left\{\begin{array}{c} 0.849*H\left(t\right)+15979.24\;\left[\Omega \right],\;\;H\geq -425\frac{A}{m}\;\;\;\\ 2.629*H\left(t\right)+16755.52\;\left[\Omega \right],\;\;H<-425\frac{A}{m} \end{array}\right.$$

**Figure 7:**
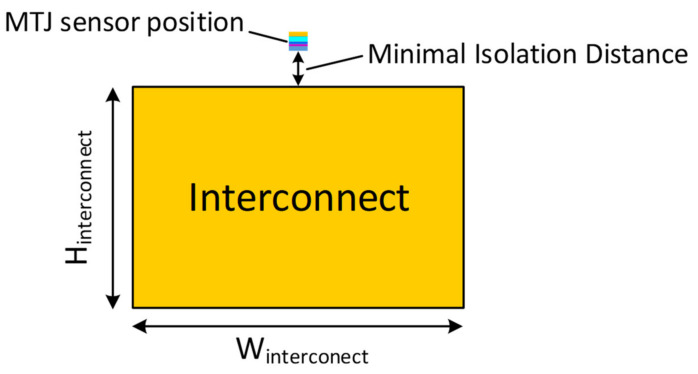
Current carrying interconnect and sensor location.

In order to determine the magnitude of the magnetic field sensed at the sensor, a simplified electromagnetic analysis of the system was conducted. First, the current density was assumed to be equally distributed over the entire surface area of the interconnect. Next, the equation was determined for the magnetic field from an infinitely thin, finite width plate a distance h beneath the sensor. Next, based on the principle of superposition, the magnetic fields due to a number of plates that were an equal distance apart from each other within the depth of the interconnect, H_interconnect_ in Figure 7, were computed. For each plate, it was assumed the current density was equal and a proportional current equal to the current magnitude divided by the number of plates. Thus, the H field could be determined mathematically, as shown in the following equations:

(7) $$\int_{c}B\cdot dl=\oint J\;\cdot dS$$

(8) $$H=\frac{B}{\mu_{0}}$$

(9) $$\int_{c}H\cdot dl=I$$

Using polar coordinates we analyze the H-field from a single plate.

(10) $$dH=\frac{I}{2*\pi *r}\;\;\hat{\theta },\;\;r=sqrt\left(y^{2}+h^{2}\right)$$

(11) $$\theta =-sin\theta \hat{y}+cos\theta \hat{z}=-\frac{h}{r}\hat{y}+\frac{y}{r}\hat{z}$$

(12) $$dH=\frac{I}{2*\pi *r}*\frac{1}{r}\left(-h\hat{y}+y\hat{z}\right)$$

(13) $$dH=\frac{I}{2*\pi *r^{2}}*\left(-h\hat{y}+y\hat{z}\right)$$

Integrating in the *y* dimension of *I* yields:

(14) $$\int dH=\int \frac{I}{2*\pi *y^{2}+h^{2}}\left(-h\hat{y}\right)$$

(15) $$H=\int \frac{1}{2\pi }\frac{I}{w}\frac{h}{h^{2}+y^{2}}dy\;$$

(16) $$H=\frac{I}{\pi *w}\tan^{-1}\left(\frac{w}{2h}\right)$$

*H* depends not only on the distance away from the interconnect, but also on the width of the wire, a finding found to be true in [43].

**Figure 8:**
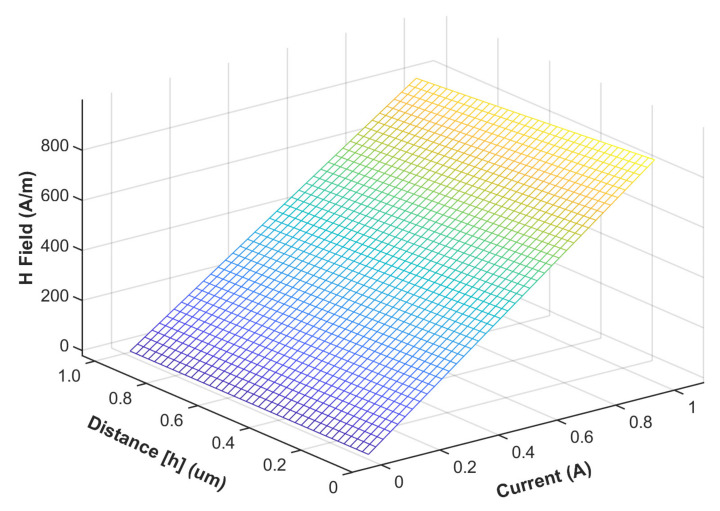
Magnetic field and current at varying sensor distances.

To find the total estimated current in the interconnect, a large, but finite, number of plates were integrated with varying distances from the first plate, which was at a distance h from the sensor to the depth of the entire interconnect, H_interconnect_. Using the minimal h distance illustrated in Figure 8, 50 nm from the surface of the interconnect, the estimated magnetic field over varying currents could then be determined.

**Figure 9:**
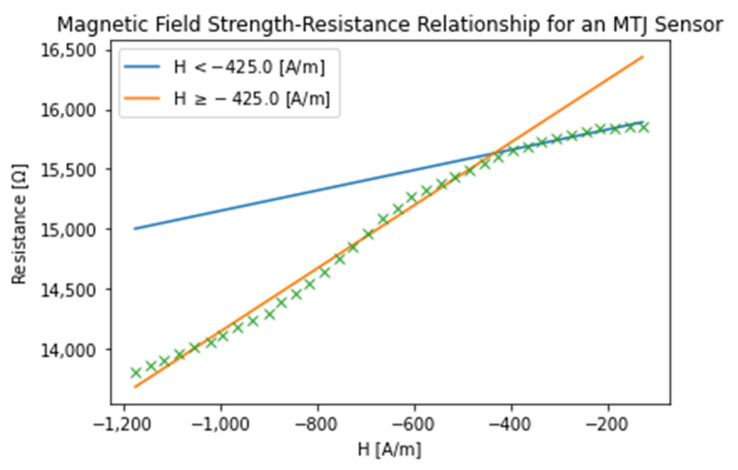
Magnetic field-resistance relationship for an MTJ Sensor.

Fitting the H-I relationship of the sensor 50 nm above the interconnect, the H-I relationship in Figure 9 was found to be approximated by the linear function:

(17) $$R=\left\{\begin{array}{c} 0.849*H\left(t\right)+15979.24\;\left[\Omega \right],\;\;H\geq -425\frac{A}{m}\;\\ 2.629*H\left(t\right)+16755.52\;\left[\Omega \right],\;\;H<-425\frac{A}{m} \end{array}\right.$$

Although empirical evidence concerning the bandwidth of our MTJ sensor was not collected, the authors of [53] indicated that while theoretical MTJ sensors have a wide bandwidth of GHz, in practice the bandwidth is closer to 100 MHz. Hence, for the simulation, we filtered the *H*-field at about a 100 MHz cutoff frequency.

(18) $$H=906.81*I\left(t\right),\;-400\;\mathrm{mA}\leq I<400\;\mathrm{mA}$$

For this particular application, DC currents on the IC trace are approximately in the range of ±400 mA. Hence, the maximum of change in resistance of this particular sensor will be ±307 Ω according to the following equations, leading to a sensitivity of around 1.9%.

(19) $$\Delta R\approx \left(\pm 400\;\mathrm{mA}*906.81*0.849\right)$$

(20) ΔR=±307 Ω

(21) $$∴\frac{\Delta R}{R}\approx \frac{307}{15979}\approx 1.9\%$$

With the small 1.9 % change in resistance, it should be noted that accurate measurements will be difficult, with potential sensing voltages through the utilization of a Wheatstone bridge of approximately 3 mV peak to peak. Because the application requires low-power ADC and a tolerable resolution, some way to boost the signal, either through an amplifier circuit or through a current-to-frequency converter or a voltage-to-time converter might be utilized to accurately measure the changing resistance, and hence, the changes in the measured current for accurate classification of the BNN. Such small-signal measurement techniques have been developed for MTJ sensor networks in the past [55]. Based on these calculations and considerations, a Verilog-A model was created to simulate the current sensing capabilities of the MTJ sensor.

### 5.2. Attacker Models and Evalution Results

The attacker scenarios developed based in the analog domain, and thus, can be detected using a reverse side-channel analysis. Hardware trojans are well-researched in literature [11] and detection with methods such as a side-channel analysis is also widely researched. However, there is little available information concerning analog hardware trojans. Hence, this study created three classes of power amplifier stage hardware trojans. The goal for these trojans was either decreasing efficiency, shutting off the device, or to inject noise in the amplification stage. Furthermore, these trojans were developed to appear to be like components found on an actual power amplifier IC and thus increase the probability of being detected. Most importantly, no matter the trojan, all were able to be classified using the lightweight BNN.

#### 5.2.1. Power Amplifier Designs

**Figure 10:**
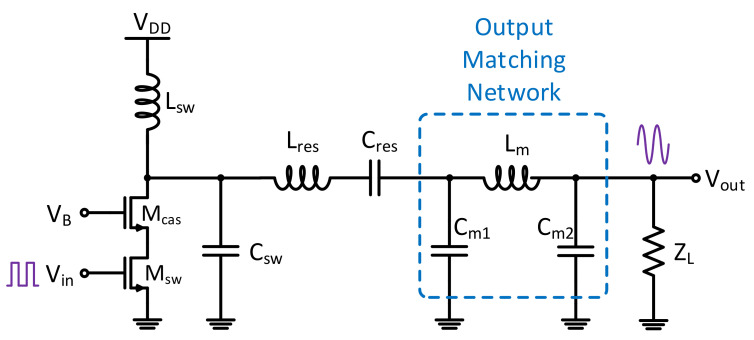
The designed Class-E PA that was used for demonstration of the proposed system.

A single-ended cascoded Class-E PA was designed and simulated for demonstration of the proposed system. By reducing the overlapping time of the transistor’s output voltage and current, power dissipation at the transistor of the switching mode PAs is minimized. Hence, supply power can be delivered to the output load more efficiently. Figure 10 shows the PA schematic that exploits the switching Class-E operation to achieve high efficiency to reflect the stringent power consumption requirements of IoT applications, and their prominent nonlinearity. A cascode transistor was added to prevent the device from breaking down. The harmonic content at the transistor drain is a result of the soft switching effect generated by C_sw_ and L_sw_. In schematic-level simulation, an output power of 18.7 to 20 dBm and a drain efficiency of 40 to 44% across process and temperature corners is achieved.

#### 5.2.2. Attacker Models on PA Designs

**Figure 11:**
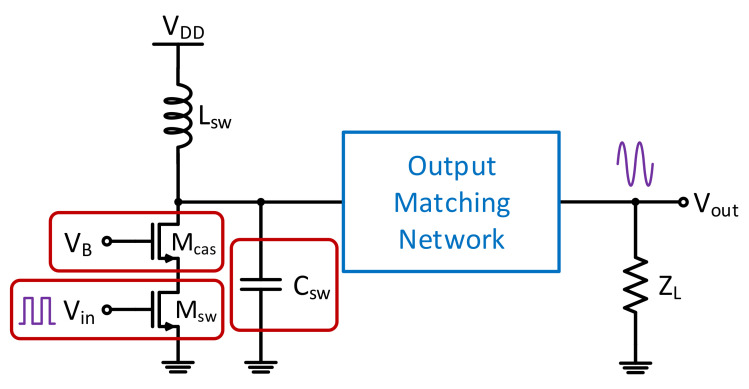
The main areas of attacker models are segmented into three categories: shut-off, parasitic capacitance, and noise injection.

The attacker models are segmented into three main categories: shut-off, parasitic capacitance, and noise injection. Few trojan models are available in literature due to the pernicious nature of trojans. Figure 11 illustrates the main areas identified in this study that can be targeted by attackers. The first area is the active device, the switching FET controlled by the input signal. Two different attacks can be carried out here, a source switch turn-off attack and a noise injection attack. A third attack can be at the output of the drain of the cascaded MOSFET, increasing the parasitic capacitance through an injected trojan capacitance circuit.

**Figure 12:**
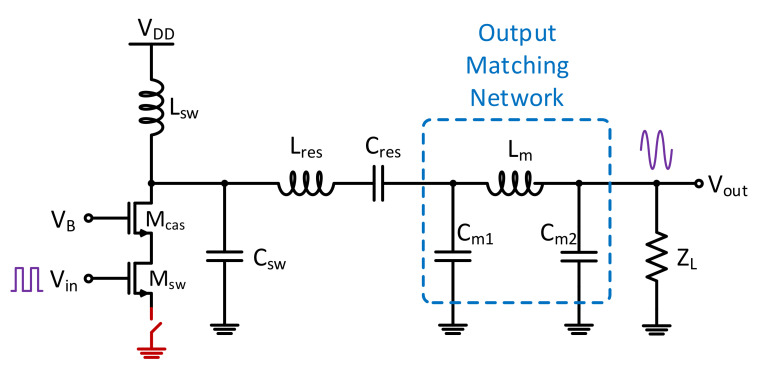
The killer switch that is used to shut off the operation of the power amplifier.

The development of trojans consisted of focusing on inserting trojans into various regions of the device and the impact of these insertions on the PA efficiency. The most obvious trojan is one that completely disables the PA. To disable the amplifier, a switch can be placed at the source of lower MOSFET that, when triggered, will cause the active devices (the transistors) to be shifted from the operation region to the off state, limiting the ability of the device to operate, as shown in Figure 12.

**Figure 13:**
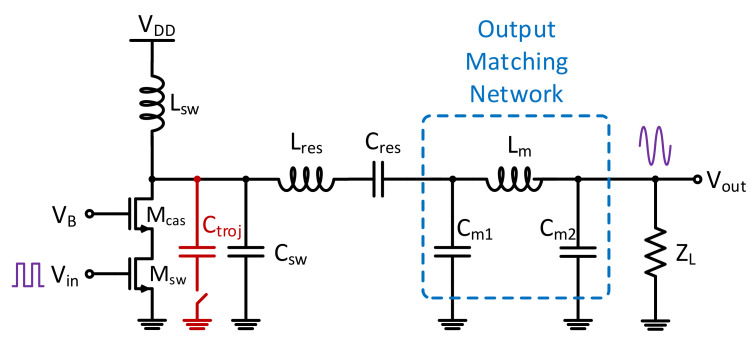
The added parasitic capacitance degrades the output efficiency and increases the power consumption of the power amplifier.

The second trojan studied was the parasitic capacitance trojan, impacting the matching network Q factor of the circuit. By increasing the capacitance on the output of the drain of the cascaded MOSFET (Figure 13), an attacker can easily cause the system to become less efficient in transmitting the input signals. The efficiency of the power amplifier determined the voltage-current relationship of the switching circuit. A Class-E amplifier is tuned to be most efficient, and thus, the drain output capacitance magnitude is carefully selected. Hence, a capacitance with a switch that can be triggered by an attacker can plausibly be fabricated on the device and be thus activated to limit the efficiency and increase the power consumption of the PA.

**Figure 14:**
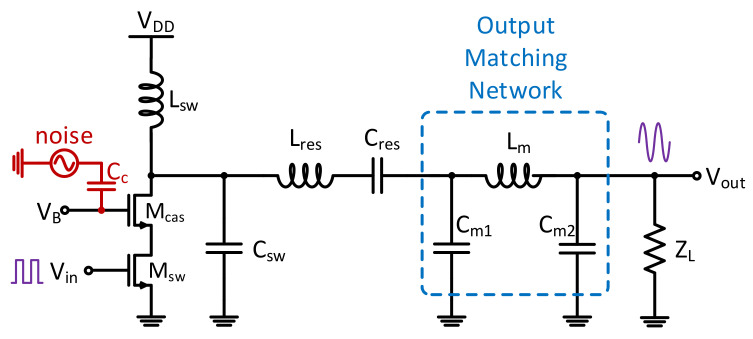
The AC-coupled noise source at the input of the cascaded MOSFET.

The third trojan studied was an AC-coupled noise source at the input of the cascaded MOSFET in the Class-E topology as in Figure 14. The radio frequency (RF) circuit designers usually set tolerances at which the amplifier can work, and hence, in moving outside of that range, the power amplifier will be less effective by coupling a noise source at the input, especially outside of that tolerance range. In this study, we analyzed noise voltages of 10% of the DC voltage and higher at frequencies equal to the input frequency.

**Figure 15:**
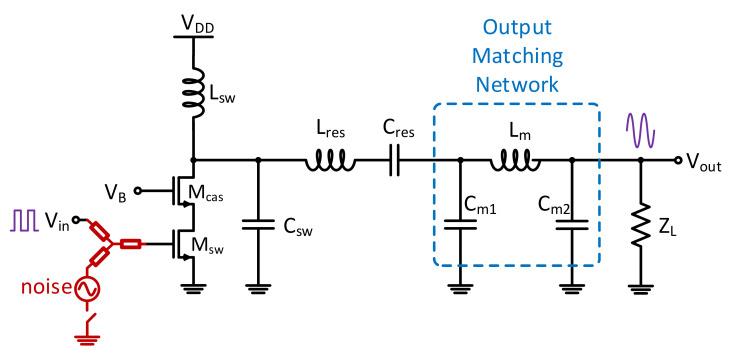
An RF power combiner to mix the noise source into the input signal.

Finally, the last trojan studied was noise injection at the input signal of the amplifier. A two-toned input, added through an RF power combiner in Figure 15, which vector-adds two analog signals together, not only causing an issue with the gain of the circuit, but also with noise in the side-band channels. By inserting a noise signal in the sideband of the of the desired signal, with the signal large enough to interfere with standard specifications, in this case the Bluetooth specification, the attacker can not only change the power consumption of the PA, but also interfere with signals in other channels as well.

Hence, all of these PA trojans were developed to change the operating ability of the Class-E power amplifier.

#### 5.2.3. Evaluation Results

All the of presented trojans and the sensor model were simulated in Cadence. The MTJ model was written in Verilog-A utilizing the current to H-field and H-field for resistance equations earlier mentioned in this study. Based on previous literature search, we also included a low-pass filter on the current-to-H-field equations at 100 MHz to approximately model the actual frequency response of the sensor. Simulated with the Class-E PA with trojans, tests were performed with a Wheatstone bridge configuration as suggested in [53], and the output went to an amplifier to allow for the determination of optimal gain for this sensing configuration.

The simulation results then were used as the input signals for the proposed BNN to classify the results. The dataset for evaluating the BNN classifier was generated by simulating the PA with various trojans in Cadence. The trojans themselves were tested by utilizing non-ideal switches that would be cycled on and off in the simulation. Each simulation was run at 1.5 V with process variations in fast-fast (FF), slow-slow (SS), and typical-typical (TT) process variations. Furthermore, the data were generated for temperatures of −40 °C, 27 °C, 60 °C, and 125 °C. Thus, for each trojan that was run, there were 12 different tests at different temperatures and process variations. For each trojan besides the switched trojan, we tested various configurations of the trojans to determine the precision of the classifier. The switched trojan only had one configuration (on and off), while the voltage tolerance trojan was swept from 0.1 V to 0.5 V in 0.1 V increments, the parasitic capacitance trojan was tested with 10 fF, 100 fF, 1 pF, and 10 pF capacitors, and the power combiner trojan was tested with combined signals of 0.024 GHz, 0.24 GHz and 2.4 GHz. All of these data for the process technologies and temperatures were combined together for each trojan in the following way: the trojan region for the source switch trojan was determined, the same length of data for that trojan was taken for each of the trojans and then were quantized at different quantization levels (4, 6, 8, 10, 12, 14, 16, and 24) between ±0.8 to produce eight different quantized test sets, and those points were then added to an overall trojan vector test vector for each quantization level that included all the different process and temperature for that particular trojan (e.g., switch, pcap 10 f, pcomb 2.4 GHz, etc.). These vectors were then added to an overall test vector that included normal operation data and all of the variations in trojans. The training sets for all the training used 20,000 points and a test set of 1000 points. Furthermore, to determine how well the classifier can resolve individual trojans, vectors that included only normal operation with a particular trojan were included. Note that in training, the dataset included an equal number of “normal” operation and “trojan” operation sets to avoid over-training the model on trojan data.

In determining the difference between a source switch circuit, a power combiner circuit, and a parasitic capacitance, the BNN performed well over all different quantization levels. The BNN was able to determine a source switch trojan with 96% accuracy over all quantization levels, a power combiner trojan with different frequencies from 200 MHz through 2.4 GHz with nearly 100% accuracy, and an approximately 85% accuracy for the parasitic capacitance trojan over all the quantization levels. When all the different types of trojans were put into the same class and compared against the “typical” signal, there was greater than 95% testing accuracy for the BNN over the various quantization levels. Table 1 summarizes the accuracies of the different type trojans.

## 6. Conclusions

Novel non-invasive sensors were developed to collect data for analysis of analog, mixed-signal, power, and EM signal behavior. To sense small changes in magnetic fields and inform the machine learning circuits, the nanoscale heterostructure was developed to be able to monolithically integrate CMOS circuits with novel spin-torque devices that can be utilized as robust high-fidelity sensors and embedded into interconnects. Lightweight learning algorithms were developed for fast threat detection at the front-end of resource-constraint devices in real time. The MTJ sessors were fabricated, measured and modeled for Cadence simulations together with the presented attacker models. The results show that the proposed system achieves 95% of the accuracy to recognize the attacker with all trojan types applied.

## Figures and Tables

**Table 1:** Accuracy summary of the different type trojans.

Trojan Type	Accuracy
Source switch trojan	96%
Parasitic capacitance trojan	85%
Noise trojan	100%
Combined trojans of all types	95%

## Data Availability

This study did not report any data.
